# EvoRSR: an integrated system for exploring evolution of RNA structural robustness

**DOI:** 10.1186/1471-2105-10-249

**Published:** 2009-08-13

**Authors:** Wenjie Shu, Ming Ni, Xiaochen Bo, Zhiqiang Zheng, Shengqi Wang

**Affiliations:** 1 Beijing Institute of Radiation Medicine, Beijing 100850, PR China; 2 College of Electro-Mechanic and Automation, National University of Defense Technology, Changsha, Hunan 410073, PR China; 3 Center for Bioinformatics, National Laboratory of Protein Engineering and Plant Genetic Engineering, College of Life Sciences, Peking University, Beijing 100871, PR China

## Abstract

**Background:**

Robustness, maintaining a constant phenotype despite perturbations, is a fundamental property of biological systems that is incorporated at various levels of biological complexity. Although robustness has been frequently observed in nature, its evolutionary origin remains unknown. Current hypotheses suggest that robustness originated as a direct consequence of natural selection, as an intrinsic property of adaptations, or as a congruent correlate of environment robustness. To elucidate the evolutionary origins of robustness, a convenient computational package is strongly needed.

**Results:**

In this study, we developed the open-source integrated system EvoRSR (Evolution of RNA Structural Robustness) to explore the evolution of robustness based on biologically important landscapes induced by RNA folding. EvoRSR is object-oriented, modular, and freely available at  under the GNU/GPL license. We present an overview of EvoRSR package and illustrate its features with the miRNA gene *cel-mir-357*.

**Conclusion:**

EvoRSR is a novel and flexible package for exploring the evolution of robustness. Accordingly, EvoRSR can be used for future studies to investigate the evolution and origin of robustness and to address other common questions about robustness. While the current EvoRSR environment is a versatile analysis framework, future versions can include features to enhance evolutionary studies of robustness.

## Background

Robustness is a fundamental and ubiquitous phenomenon in biological systems, in which phenotypes are resistant to change in the presence of various perturbations. When these perturbations are inherited, such as genetic mutations, the phenomenon is known as genetic robustness. Alternatively, when the perturbations are due to environmental factors, the phenomenon is called environmental robustness [1]. Both types of robustness appear at various levels of biological organization, affecting gene expression, protein folding, metabolic flux, physiological homeostasis, development, and organism fitness [2]. Biologists' long-standing interest in robustness has roots in Fisher's work on dominance [3-5] and Waddington's developmental canalization research [6,7]. Despite being found throughout nature, the evolutionary origins of robustness remain unclear. Current competing explanations for the origins of robustness include that it evolves as a direct consequence of natural selection, as an intrinsic property of adaptations, or as congruent correlate of environment robustness. Additionally, it is unknown how robustness evolves and how the robustness varies along the Hamming distance from the WT sequence.

Addressing these questions requires a convenient computational package that will fully elucidate the evolutionary origins of robustness. A good example to study for clarifying the origins of robustness is RNA folding from sequences into secondary structures. RNA folding provides a convenient biophysical model of a genotype-phenotype map that has been used in studies for robustness, evolvability, and epistasis. In such studies, RNA folding can be precisely defined and statistically measured, revealing simultaneous and non-independent effects of natural selection [8,9]. These studies have focused on the robustness of RNA folding in viruses [10-12], viroids [13,14], and microRNAs [15-18].

Given a quantitative measure of structural robustness [15,18,19], we developed an integrated system named EvoRSR (Evolution of RNA Structural Robustness) to explore the evolution of robustness based on important landscapes induced by RNA folding. EvoRSR is object-oriented, modular in design and freely available at  under the GNU/GPL license. This open-source package inspects the evolution and origin of robustness through sampling genotype (sequence) space at each Hamming distance from the WT sequence. Here, we describe the EvoRSR package and analyze the miRNA gene *cel-mir-357 *to illustrate how EvoRSR works.

## Implementation

### Mechanism and workflow of EvoRSR

**Figure 1:**
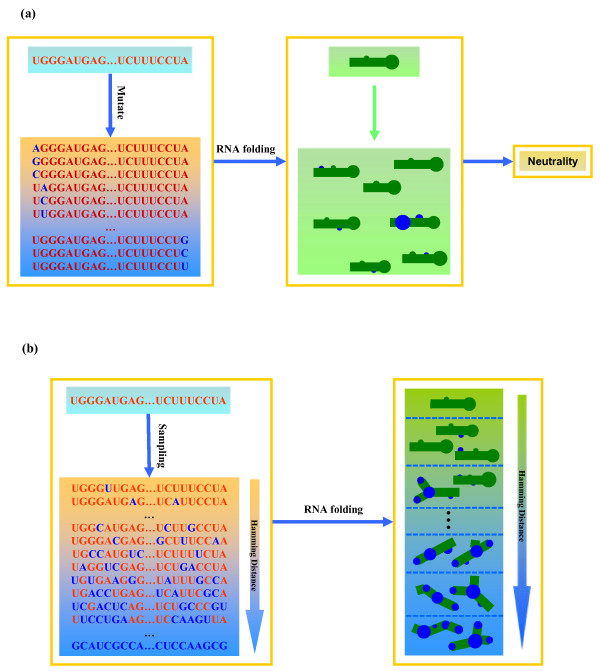
**The mechanism of EvoRSR**. (a) Evaluation of genetic robustness. (b) Sampling and folding on genotype space at each Hamming distance.

Figure 1 illustrates the mechanism of EvoRSR. EvoRSR studies the evolution of robustness based on landscapes that result from mapping micro-configurations to scalar or nonscalar entities. Here, the micro-configurations are sequences of nucleotides. The scalar properties include free-energy of secondary structure and neutrality. Free-energy of secondary structure describes the thermodynamic stability of RNA secondary structure (conferring environmental robustness) [15,16,19]. Neutrality (see Figure 1a) quantitatively measures the genetic robustness of RNA secondary structure [15,16,18,19]. Based on these two scalar properties, we defined the free-energy landscape and neutrality landscape, respectively. The nonscalar structure landscape is generated from the RNA secondary structure. Based on these three landscapes, EvoRSR investigates the evolution of robustness in the phenotype space by sampling on genotype (sequence) space at each Hamming distance from the WT RNA sequence (see Figure 1b).

**Figure 2:**
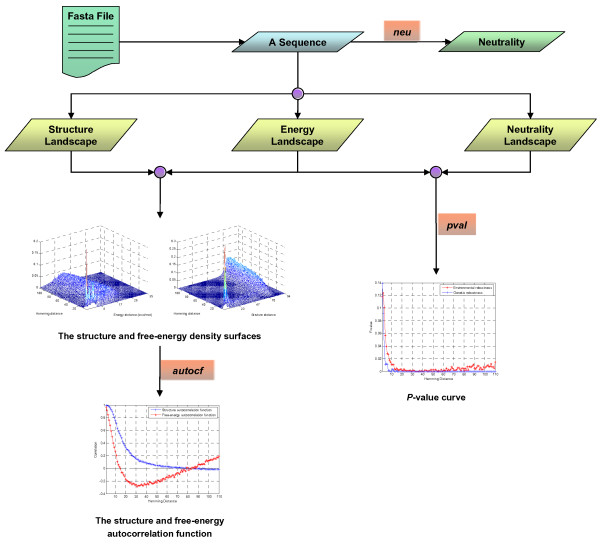
**The workflow of EvoRSR**.

The EvoRSR package is a free package written in C, which runs in a command-line mode within a Linux/Unix environment. The Vienna RNA package [20] is required to run the program. Detailed installation instructions for EvoRSR are provided on its web site. Currently, three programs are included in this package. Figure 2 shows the workflow of EvoRSR.

### Evaluation of genetic and environmental robustness

Formally, the neutrality η of an RNA sequence with length *l *is defined as



where *d *is the base-pair distance between the secondary structures of the WT sequence and its mutant, averaged over all 3 × *l *one-mutant neighbors. *d *is calculated by RNADISTANCE in Vienna RNA package [21]. Thus, η represents the average fraction of the structure that remains unchanged after a mutation occurs. The free-energy, *dG*, is quantitatively measures the thermodynamic stability (which confers environmental robustness) of a WT RNA sequence [15-17,19]. *dG *is calculated as the minimum free-energy of secondary structure obtained by RNAFOLD in Vienna RNA package [21]. In the EvoRSR package, *Evoneu *is applied to calculate the η *s *and *dGs *of the sequences in a Fasta file (see Figure 2).

Because RNA molecules may function in dynamical, structural reconfigurations [22,23], an RNA molecule is better described by an ensemble of secondary structures, that have free energies close to the minimum of free-energy. In this case, we revise the quantitative definition of genetic and environmental robustness. The base-pair distance *d *in equation (1) is replaced by the general multi-structure distance between the ensemble of secondary structures of the WT sequence and its mutant [24], and the minimum free-energy *dG *is replaced by the ensemble free-energy.

### Landscape and its density surfaces

For each WT RNA sequence, we employ a Monte Carlo method to sample sequences in the genotype space at each Hamming distance from the WT RNA sequence. The set of total sampling sequences is denoted by *S*, which can be divided into subsets *S*_*i*_, *i *= 1, 2, ..., *l *that represent the set of sampling sequences within a Hamming distance of *i *from the WT sequence. All the subsets have an identical size, (|*S*_*i*_| = *N*, *i *= 1, 2, ..., *l*).

As a generic tool for the study statistical properties of landscapes, we propose the use of a two-dimensional probability density surface [25,26]. A density surface *P(t|h) *is the conditional probability that given two sequences Hamming distance *h *from each other, the two configurations have either a base-pair distance *t *or a free-energy difference *t*. The density surface describes how the distributions of free-energy values and configuration differences change along the Hamming distance from the WT sequence. Furthermore, the density surface condenses statistical aspects of the correlation between sequences and structures and provides a tool to derive and calculate local and global properties of sequence-structure relations.

### Autocorrelation function and correlation length

Landscape can be characterized statistically by autocorrelation functions [27,28], which can be expressed in terms of mean squared distance:



⟨*d*^2^⟩ is the mean squared distance sampled over the entire sequence space, and  is the conditional mean squared distance. Autocorrelation functions of base-pair distances ρ(*h*) are approximated by an exponential fit to calculate a correlation length ℓ for secondary structures in sequence space:



The correlation length increases roughly with the sequence length *l *[25]. Autocorrelation functions and correlation lengths of structures characterize the sequence-structure relation by a single function or a single value, respectively. They provide a useful measure for the sensitivity of RNA structures against point mutations. In the EvoRSR package, they are computed by the program *Evoautocf *(see Figure 2).

### *P*-value curve of robustness

For each WT RNA sequence, EvoRSR measures the neutrality of the WT sequence, *η*_*WT*_, and evaluates the neutralities , *i *= 1, 2, ..., *l*, *j *= 1, 2, ..., *N *of the corresponding sampling sequences in *S*_*i*_, *i *= 1, 2, ..., l,. To evaluate the level of the increased neutrality for each WT sequence at each Hamming distance separately, the rank of the neutrality of WT sequence, *r*_*i *_*i *= 1, 2, ..., l, among the neutralities of the sampling sequences in *S*_*i*_, *i *= 1, 2, ..., l, is calculated. This order statistics measure has no requirements on the nature of the neutrality value distribution. The significance level of robustness of WT sequence at each Hamming distance is then defined as the *P*-value curve , *i *= 1, 2, ..., *l*, which estimates the probability of observing an equal or higher neutrality value by chance at each Hamming distance. The same analysis applies to the environmental robustness, in which the neutrality of a WT RNA sequence is replaced by its free-energy, *dG*. The significance analysis process is realized by the program *Evopval *in the EvoRSR package (see Figure 2).

## Results and discussion

To illustrate how EvoRSR can be used to study the evolution of robustness, we analyzed the *C. elegans *miRNA *mir-357 *(see Figure 2). The detail results are presented as Additional file available on the website of EvoRSR [see Additional file 1]. Our result indicates that along the Hamming distance from the WT sequence the genetic and environmental robustness of miRNA gene *cel-mir-357 *vary in a consistent way, and the sub-optimal structures may have little effect on our conclusions [see Additional file 1].

Robustness reduces an organism's susceptibility to genetic and environmental perturbations. To understand the evolutionary origins of robustness, we needed to know how phenotype and genotype are related, and how the genotype-phenotype map interacts with evolution. We developed a convenient computational package EvoRSR to fully elucidate the evolutionary mechanisms of the genetic robustness in RNA structure. EvoRSR can investigate the statistical details of RNA structure and the free-energy landscapes, providing the corresponding autocorrelation function and correlation length. Based on these landscapes, EvoRSR explored the evolution of genetic robustness along the Hamming distance from the WT sequence. By providing the *P*-value curves of both genetic and environmental robustness, EvoRSR presents a scenario of how, and how fast, significant levels of robustness vary along the Hamming distance from the WT sequence. Additionally, EvoRSR helped examine the statistical relationship between genetic and environmental robustness along the Hamming distance from the WT sequence.

EvoRSR is a novel and flexible package for exploring the evolution of genetic robustness. EvoRSR was used to study the robustness of RNA secondary structures, providing a promising framework to examine central issues concerning the evolution of robustness [15,16]. Recently, we examined the neutrality of the structural element in 1,082 native miRNA genes from six species and demonstrated that the structural elements within native miRNA genes exhibited a significantly higher level of genetic robustness [18]. An examination of miRNAs of several eukaryotic species revealed that the stem-loop structures of miRNA genes exhibits a significantly higher level of genetic robustness compared to randomly reshuffled pseudo miRNAs [15,16]. This finding indicated that the excess robustness of miRNAs goes beyond the intrinsic robustness of the stem-loop structure. Our results indicate that the increased genetic robustness of miRNAs may result from congruent evolution for environment robustness [16]. However, Borenstein and Ruppin suggested that the excess robustness of miRNA stem-loops results from direct evolutionary pressure for increased robustness [15]. Furthermore, these studies do not solve how both genetic as well as environmental robustness evolve or how environmental and genetic robustness correlate with each other along the evolutionary path from the WT sequence. EvoRSR will elucidate the evolutionary mechanisms of genetic robustness.

While the EvoRSR environment is a versatile analysis framework already in the present version, there have many options for further enhancement. The mechanisms underlying robustness are diverse, ranging from thermodynamic stability at the RNA and protein level to behavior at the organismal level [2]. The increased neutrality and thermodynamic stability of RNAs examined by EvoRSR can be conceived as first-order robustness, based only on RNA folding map that that assigns each sequence to a minimum-free-energy structure. The simplicity of this form of robustness, the full tractability of RNA secondary structure, and the complete control of reference background facilitate the exploration of its evolutionary origins. Protein structures, a step up in complexity, may possess similar features to test the evolution of robustness. With a better understanding of protein folding and more accurate prediction algorithms [29], our methodology can be applied to the evolution of robustness in protein structures. Based on the understanding of the first-order robustness, we can further explore the evolution of higher-level robustness.

## Conclusion

In this study, we developed the open-source integrated system EvoRSR (Evolution of RNA Structural Robustness) to explore the evolution of robustness based on biologically important landscapes induced by RNA folding. EvoRSR is object-oriented, modular, and freely available at  under the GNU/GPL license. EvoRSR can be used for future studies to investigate the evolution and origin of robustness and to address other common questions about robustness. While the current EvoRSR environment is a versatile analysis framework, future versions can include features to enhance evolutionary studies of robustness.

## Availability and requirements

**Project name**: EvoRSR (Evolution of RNA Structural Robustness)

**Project home page**: 

**Operating system(s)**: Linux, UNIX (no GUI)

**Programming language**: C++ and Perl

**Other requirements**: Vienna RNA package

**License**: GNU/GPL license

**Restrictions to use by non-academics**: None

## Competing interests

The authors declare that they have no competing interests.

## Authors' contributions

WS and MN wrote the programs, analyzed the results. WS drafted the manuscript. XB and ZZ helped in analysis and discussion, gave useful comments. SW and XB guided the project. All authors read and approved the final manuscript.

## Supplementary Material

Additional file 1 **The results of *cel-mir-357***. The *C. elegans *microRNA (miRNA) *mir-357 *(*cel-mir-357*) is analyzed as example to illustrate how EvoRSR can be helpful for studying the evolution of robustness. Click here for file
