# Supplementary material for: EvoRSR: an integrated system for exploring evolution of RNA structural robustness
Source: BMC Bioinformatics. 2009 Aug 13;10:249. doi: 10.1186/1471-2105-10-249 (PMC2731758; doi:10.1186/1471-2105-10-249)
Supplement: Additional file 1 — The results of cel-mir-357. The C. elegans microRNA (miRNA) mir-357 (cel-mir-357) is analyzed as example to illustrate how EvoRSR can be helpful for studying the evolution of robustness. [file 1471-2105-10-249-S1.pdf]

Supplementary materials for

EvoRSR: an integrated system for exploring evolution of RNA structural robustness

Wenjie Shu[1,2†], Ming Ni [1,3†], Xiaochen Bo[1*], Zhiqiang Zheng[2], Shengqi Wang[1*]

[1]Beijing Institute of Radiation Medicine, Beijing 100850, China
[2]College of Electro-Mechanic and Automation, National University of Defense Technology, Changsha, Hunan 410073, China
[3]Center for Bioinformatics, National Laboratory of Protein Engineering and Plant Genetic Engineering, College of Life Sciences, Peking University, Beijing 100871, China.

[†]The authors wish it to be known that, in their opinion, the first two authors should be regarded as joint First Authors.

[*]To whom correspondence should be addressed. Tel & Fax: +86 10 68210077 66932211; Email: sqwang@bmi.ac.cn. Correspondence may also be addressed to Xiaochen Bo. Tel & Fax: +86 10 68210077 66931422; Email: boxc@bmi.ac.cn.

Email addresses:

WS: shuwj@bmi.ac.cn
MN: nim@mail.cbi.pku.edu.cn
XB: boxc@bmi.ac.cn
ZZ: xyzheng@sohu.com
SW: sqwang@bmi.ac.cn

Results

To illustrate how EvoRSR can be helpful for studying the evolution of robustness, the *C. elegans* microRNA (miRNA) *mir-357* (*cel-mir-357*) is analyzed as example. miRNAs are abundant endogenous ~22-nucleotide (nt) noncoding RNAs, which are cleaved from ~70 nt precursors (pre-miRNA) that fold into a stem-loop hairpin structure, through the action of Dicer endonuclease [1-3].

We first focus on the local and global features of the structure and the free-energy landscapes. The structure as well as free-energy landscapes of *cel-mir-357* are generated by EvoRSR, and the corresponding density surfaces are illustrated in Figure S1. Although for very small Hamming distances ($h = 1, 2, 3$) the most probable structures are identical or very similar, there is none the less some probability that even a single mutation substantially alters the structure (see Figure S1a). Furthermore, it is extremely unlikely that two randomly chosen sequences fold into identical structures. This is in sharp contrast to the free-energy landscape (see Figure S1b), indicating that there are many more structures than energies. Nonetheless, the distribution of structure distances approaches already, at fairly short Hamming distances, the distribution expected for random sequences, indicating that the structures of *cel-mir-357* sequence and its mutants at that distance or larger are effectively uncorrelated. Our data suggests that memory of *cel-mir-357* is sufficiently lost to allow the mutants at that distance to acquire any frequent minimum free-energy structure, at least in its essential features.

Although the landscape present us a full view about how, and how fast, the distributions of the configuration and free-energy differences vary as the sequences become more and more uncorrelated with increasing Hamming distance from the WT sequence, we still should derive and calculate its local and global properties. We investigate the statistical features of both types of landscapes by computing the autocorrelation functions, as well as correlation length, as a function of distance in sequence space. From the density surface of landscape the complete autocorrelation function can be recovered [4]. This function is to a reasonable approximation a single decaying exponential with a correlation length, ℓ. Figure S2 shows the structure and free-energy

autocorrelation functions $\rho(h)$ versus h for *cel-mir-357*. For *cel-mir-357*, both the structure and free-energy autocorrelation functions decay rapidly with the Hamming distance h (see Figure S2). They drop to one-half of their initial values within a few mutations. There is very little correlation between configurations on the landscape in the range $h > l/3$. The result shows that both the structure and free-energy landscapes should have exponentially decaying autocorrelation functions independent of the details of neighborhood assignment.

Based on the landscapes, we examine the significance levels of both genetic as well as environmental robustness at each Hamming distance, to see how the significance levels of robustness of both kinds vary as the sequences become more and more uncorrelated with increasing Hamming distance from the WT sequence. Figure S3 shows the *P*-value curves of genetic and environmental robustness of *cel-mir-357*. Even for very small Hamming distances ($h = 1,2,3$), the significance level of genetic robustness is not larger 0.15. Beyond distance $h = 3$, *cel-mir-357* shows significant genetic robust at each Hamming distance ($p_i < 0.05, i = 4,5,\ldots,l$), and in the range $h > 15$, the significance level becomes independent of h ($p_i = 0, 15 < i \le l$). Additionally, examining the significance level of the environmental robustness of *cel-mir-357* at each Hamming distance provides a similar picture as that of genetic robustness. For miRNA *cel-mir-357*, the significance levels of both genetic as well as environmental robustness decrease as a function of the mutational distance from the WT.

To further explore the evolution of genetic robustness, EvoRSR examines the statistical relationship between genetic and environmental robustness along the Hamming distance for miRNA gene *cel-mir-357*. The statistical analysis is performed for the paired values of the *P*-values of genetic and environmental robustness at each Hamming distance. For *cel-mir-357*, the Pearson's correlation coefficient is 0.8893 ($p = 1.765 \times 10^{-38}$), indicating that the correlation between the genetic and environmental robustness is very high. Our result indicates that along the Hamming distance from the WT sequence the genetic and environmental robustness of miRNA gene *cel-mir-357* vary in a consistent way.

To mitigate the uncertainty of secondary structure prediction, sub-optimal structures can be optionally taken into account. In this case, a WT RNA sequence is described by an ensemble of secondary structures in the EvoRSR package. Using the revised quantitative measures of both genetic and environmental robustness (see Methods), we re-analyze the miRNA gene *cel-mir-357* using EvoRSR. The results indicate that the sub-optimal structures may have little effect on our conclusions (supplementary Figure S4 ~ S6). However, the computational time in this case is much longer.

References

1. Hutvagner G, McLachlan J, Pasquinelli AE, Balint E, Tuschl T, Zamore PD: **A cellular function for the RNA-interference enzyme Dicer in the maturation of the let-7 small temporal RNA**. *Science* 2001, **293:**834-838.

2. Lee RC, Feinbaum RL, Ambros V: **The C. elegans heterochronic gene lin-4 encodes small RNAs with antisense complementarity to lin-14**. *Cell* 1993, **75:**843-854.

3. Ketting RF, Fischer SE, Bernstein E, Sijen T, Hannon GJ, Plasterk RH: **Dicer functions in RNA interference and in synthesis of small RNA involved in developmental timing in C. elegans**. *Genes Dev* 2001, **15:**2654-2659.

4. Fontana W, Konings DA, Stadler PF, Schuster P: **Statistics of RNA secondary structures**. *Biopolymers* 1993, **33:**1389-1404.

Figure legends

Figure S1. The density surface of landscape.

At each Hamming distance from *cel-mir-357*, $N = 1,000$ sequences are randomly sampled. The structure density surface (a) and the free-energy density surface (b) of *cel-mir-357* are obtained by evaluating the distributions of the configuration and free-energy differences between the WT sequence (*cel-mir-357*) and the sampling sequences at each Hamming distance, respectively.

Figure S2. The landscape autocorrelation functions.

The structure autocorrelation function (blue line) and free-energy autocorrelation function (red line) for *cel-mir-357*.

Figure S3. The *P*-value curves of genetic and environmental robustness.

At each Hamming distance from WT *cel-mir-357* equence, the neutrality η_{WT} and free-energy dG_{WT} of *cel-mir-357* is compared with that of the $N = 1,000$ sampling sequences. The *P*-value curves of genetic robustness (blue line) and environmental robustness (red line) of a specific significantly robust miRNA gene (cel-mir-357).

Figure S4. The density surface of landscape for a specific significantly robust miRNA gene (cel-mir-357) in the case of considering sub-optimal structures.

In the case of considering sub-optimal structures, a WT RNA sequence is better described by an ensemble of secondary structures. The base-pair distance d in the definition of neutrality is replaced by the general multistructure distance, and the minimum free-energy is replaced by the ensemble free-energy, respectively. Using the revised definition of neutrality and free-energy, we re-plot the structure density surface (a) and the free-energy density surface (b) of cel-mir-357.

Figure S5. The landscape autocorrelation functions in the case of considering sub-optimal structures.

In the case of considering sub-optimal structures, the structure autocorrelation function (blue line) as well as free-energy autocorrelation function (red line) for cel-mir-357.

Figure S6. The *P*-value curves of genetic and environmental robustness in the case of considering sub-optimal structures.

In the case of considering sub-optimal structures, the *P*-value curves of genetic robustness (blue line) as well as environmental robustness (red line) for cel-mir-357.

Figure S1

(a)

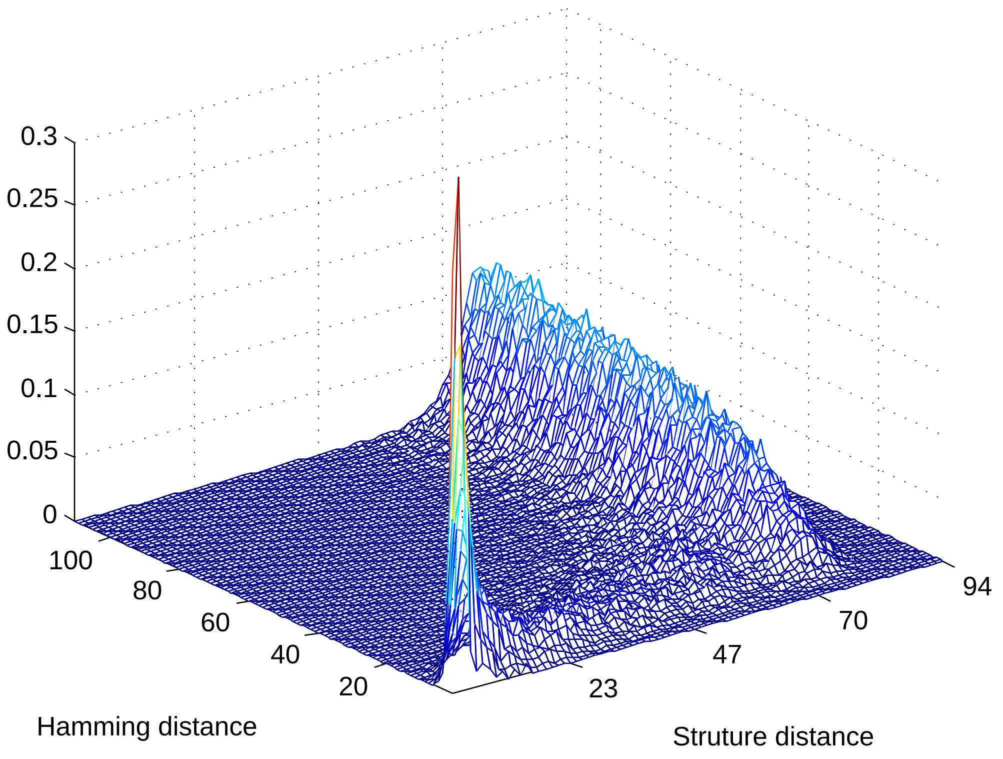
0.3
0.25
0.2
0.15
0.1
0.05
0
100
80
60
40
20
Hamming distance
23
47
70
94
Struture distance

(b)

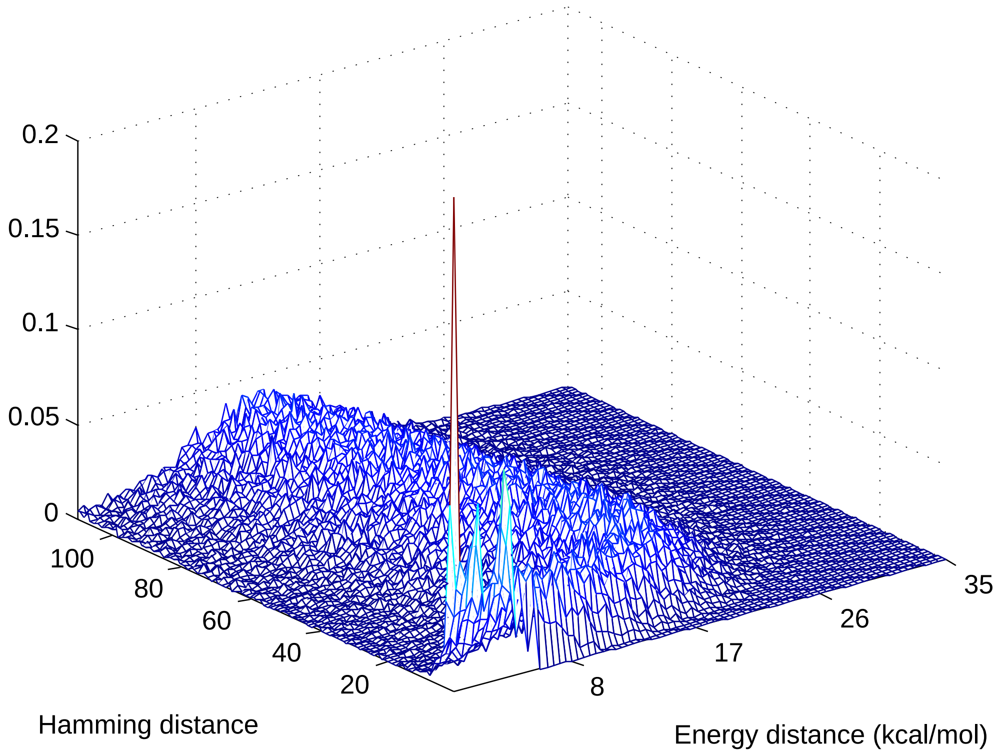
0.2
0.15
0.1
0.05
0
100
80
60
40
20
Hamming distance
8
17
26
35
Energy distance (kcal/mol)

Figure S2

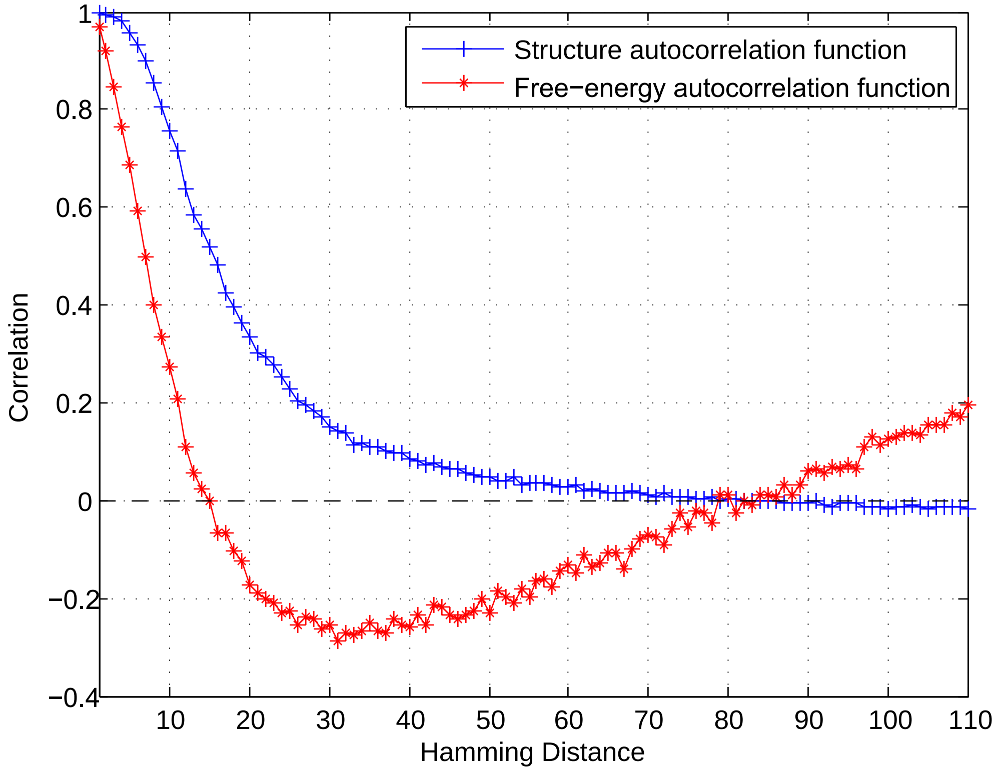

Structure autocorrelation function
Free−energy autocorrelation function
Correlation
Hamming Distance
1
0.8
0.6
0.4
0.2
0
−0.2
−0.4
10
20
30
40
50
60
70
80
90
100
110

Figure S3

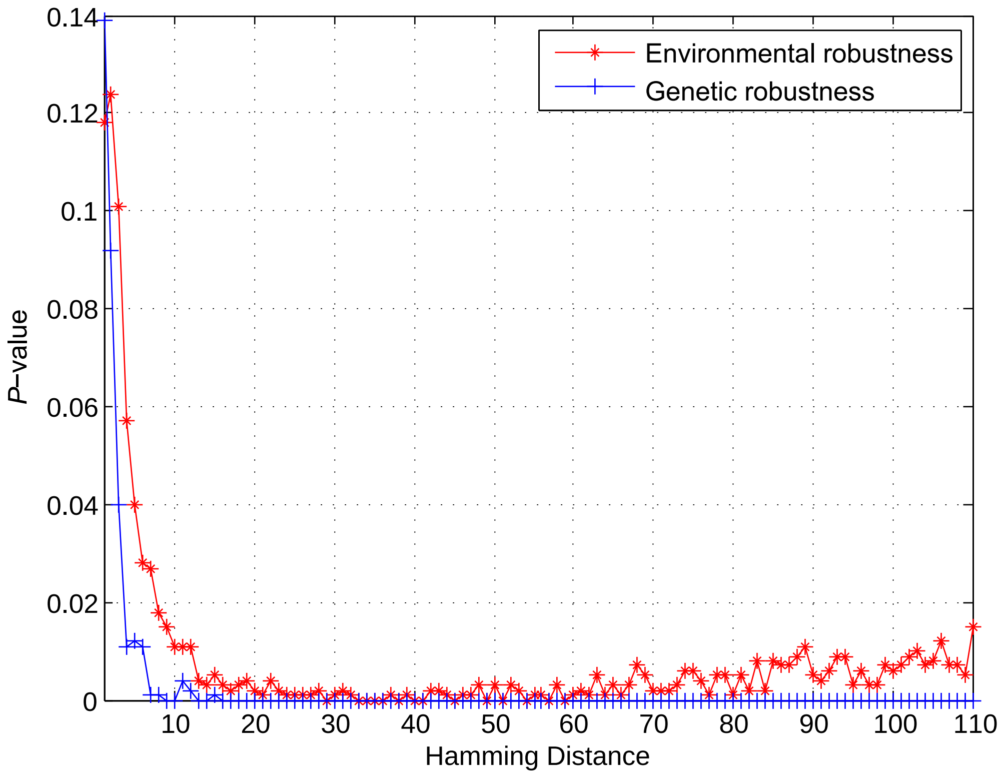

Environmental robustness
Genetic robustness
P−value
Hamming Distance
0
0.02
0.04
0.06
0.08
0.1
0.12
0.14
10
20
30
40
50
60
70
80
90
100
110

Figure S4

(a)

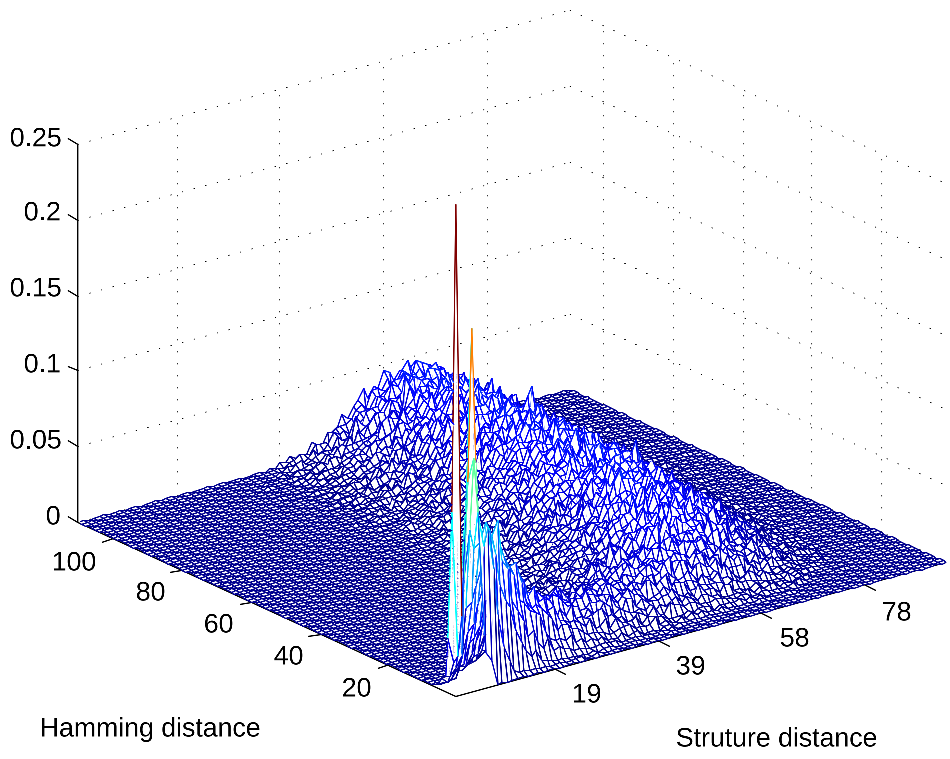
0.25
0.2
0.15
0.1
0.05
0
100
80
60
40
20
Hamming distance
19
39
58
78
Struture distance

(b)

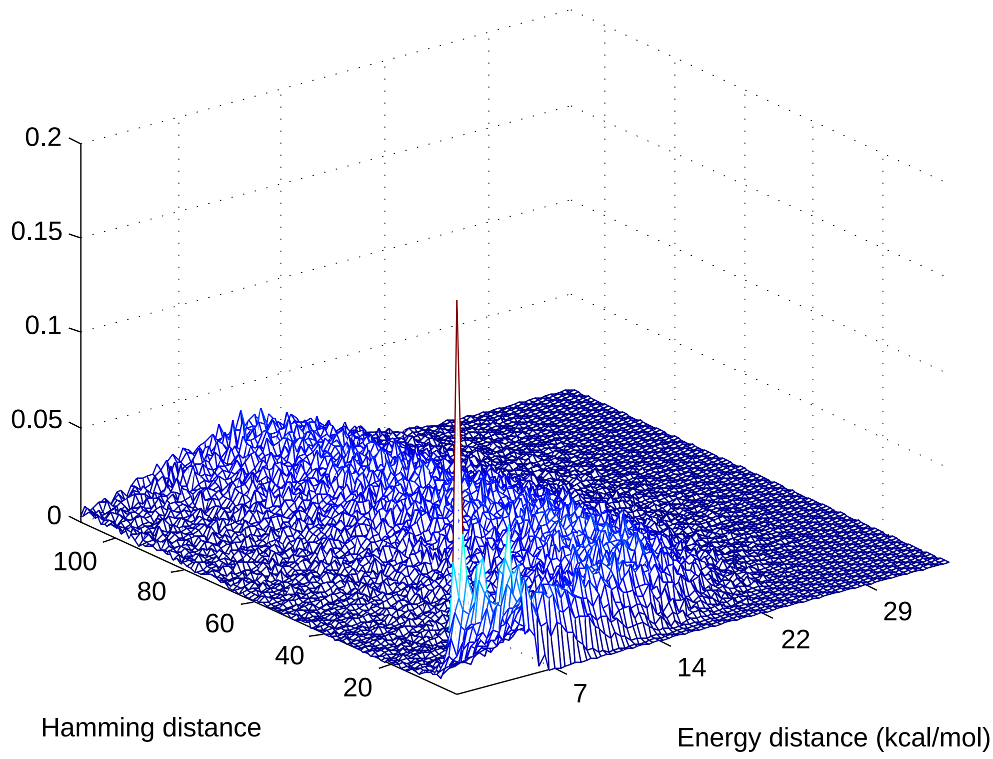
0.2
0.15
0.1
0.05
0
100
80
60
40
20
Hamming distance
7
14
22
29
Energy distance (kcal/mol)

Figure S5

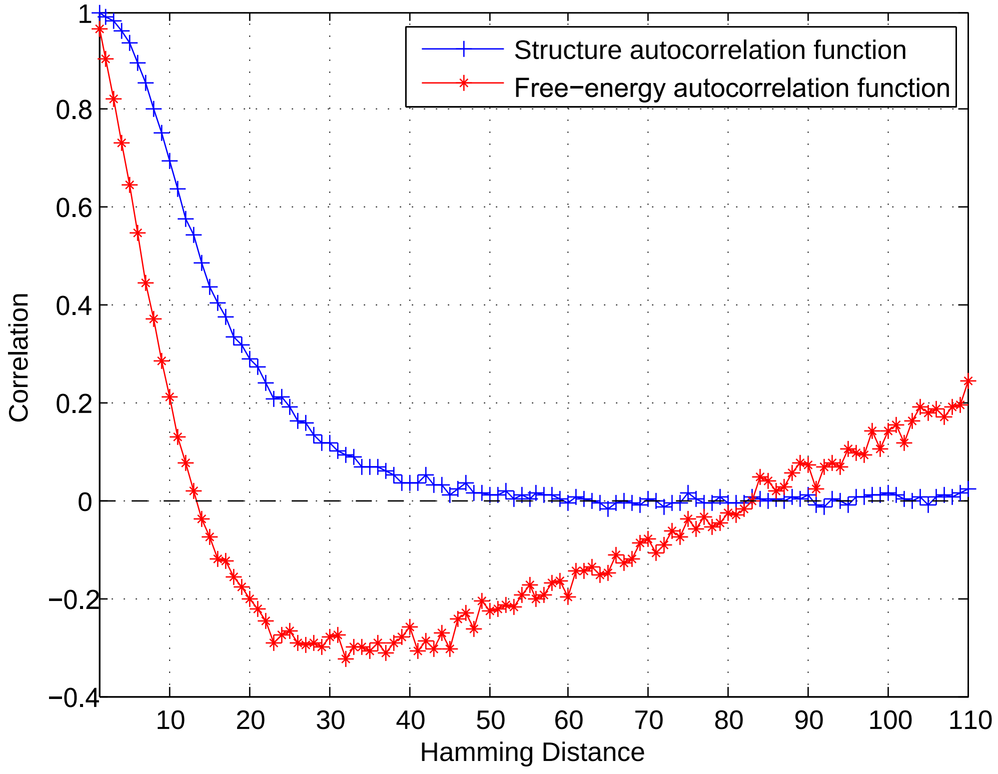

Structure autocorrelation function
Free−energy autocorrelation function
Correlation
Hamming Distance
1
0.8
0.6
0.4
0.2
0
−0.2
−0.4
10
20
30
40
50
60
70
80
90
100
110

Figure S6

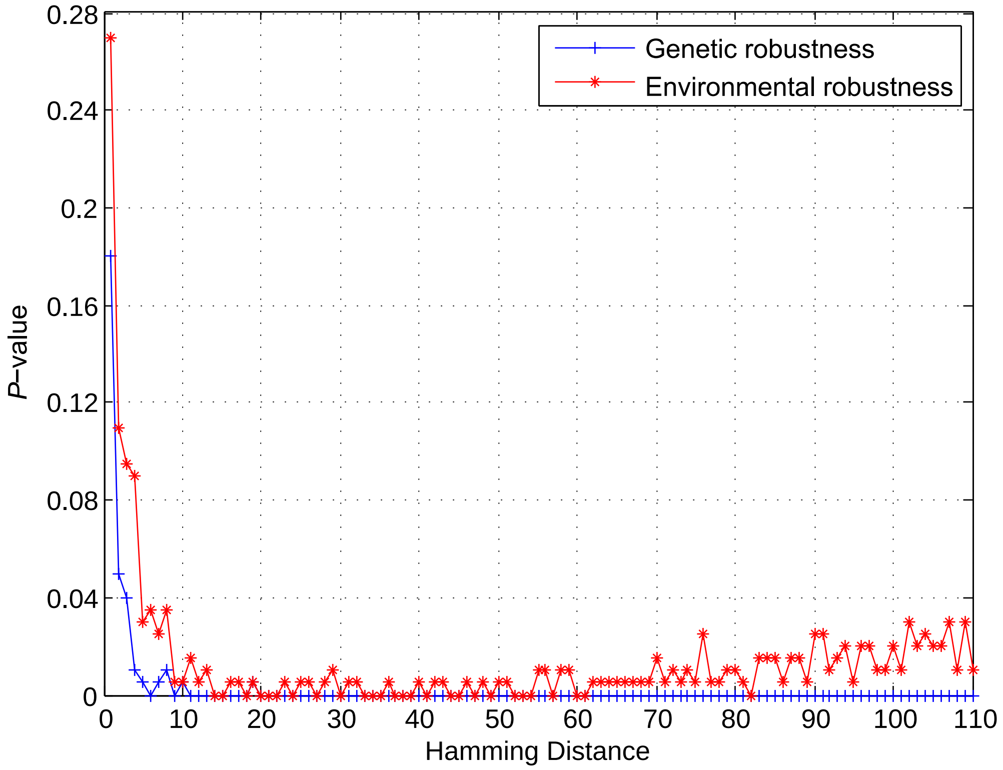

Genetic robustness
Environmental robustness
P−value
Hamming Distance
0.28
0.24
0.2
0.16
0.12
0.08
0.04
0
0
10
20
30
40
50
60
70
80
90
100
110
